# Long-Term Effects of Chronic Oral Ritalin Administration on Cognitive and Neural Development in Adolescent Wistar Kyoto Rats

**DOI:** 10.3390/brainsci2030375

**Published:** 2012-09-12

**Authors:** Margery C. Pardey, Natasha N. Kumar, Ann K. Goodchild, Kelly J. Clemens, Judi Homewood, Jennifer L. Cornish

**Affiliations:** 1 Department of Psychology, Macquarie University, Sydney 2109, Australia; Email: margery.pardey@mq.edu.au (M.C.P.); k.clemens@unsw.edu.au (K.J.C.); judi.homewood@mq.edu.au (J.H.); 2 The Australian School of Advanced Medicine, Macquarie University, Sydney 2109, Australia; Email: nnk4n@virginia.edu (N.N.K.); ann.goodchild@mq.edu.au (A.K.G.)

**Keywords:** methylphenidate, adolescent, tyrosine hydroxylase, chronic administration, prefrontal cortex, impulsivity, impulsive choice

## Abstract

The diagnosis of Attention Deficit Hyperactivity Disorder (ADHD) often results in chronic treatment with psychostimulants such as methylphenidate (MPH, Ritalin^®^). With increases in misdiagnosis of ADHD, children may be inappropriately exposed to chronic psychostimulant treatment during development. The aim of this study was to assess the effect of chronic Ritalin treatment on cognitive and neural development in misdiagnosed “normal” (Wistar Kyoto, WKY) rats and in Spontaneously Hypertensive Rats (SHR), a model of ADHD. Adolescent male animals were treated for four weeks with oral Ritalin^®^ (2 × 2 mg/kg/day) or distilled water (dH_2_O). The effect of chronic treatment on delayed reinforcement tasks (DRT) and tyrosine hydroxylase immunoreactivity (TH-ir) in the prefrontal cortex was assessed. Two weeks following chronic treatment, WKY rats previously exposed to MPH chose the delayed reinforcer significantly less than the dH_2_O treated controls in both the DRT and extinction task. MPH treatment did not significantly alter cognitive performance in the SHR. TH-ir in the infralimbic cortex was significantly altered by age and behavioural experience in WKY and SHR, however this effect was not evident in WKY rats treated with MPH. These results suggest that chronic treatment with MPH throughout adolescence in “normal” WKY rats increased impulsive choice and altered catecholamine development when compared to vehicle controls.

## 1. Introduction

Attention Deficit/Hyperactivity Disorder (ADHD) is the most common neurobehavioural childhood disorder [1]. The psychostimulant methylphenidate (MPH; Ritalin^®^) is widely prescribed and effectively reduces the ADHD symptoms of hyperactivity, impulsivity and inattention [2]. However, diagnosis of ADHD relies heavily on subjective interpretations of the diagnostic criteria [3]. Primary care physicians vary greatly in their assessment methods and do not always follow “best practice” guidelines when diagnosing ADHD [4]. Such variations in diagnostic procedures would likely increase misdiagnosis of the disorder [5]. It has been reported that stimulants, such as MPH, are administered to children who do not meet full diagnostic criteria of ADHD [6,7] and also to children as young as 2 years of age [8]. 

Inappropriate drug treatment throughout childhood and adolescence could have long-term effects on brain development. The human nervous system undergoes synaptogenesis and myelination through puberty, with continued remodelling of neural circuitry (synaptic plasticity) during adulthood [9]. Similar periods of neural development are evident in the rat brain, with the duration of this period reduced to days or weeks in the rat, compared to months or years in humans [9]. 

The pharmacological action of MPH is to increase synaptic levels of catecholamines by blocking their re-uptake at dopamine (DA) and noradrenaline (NA) transporters [10,11,12]. Therapeutic doses of MPH administered to rats increase both NA and DA in the prefrontal cortex (PFC) [13,14]. The PFC is involved in higher cognitive functions. Clear distinctions have been made between the functions of the medial and orbital regions of the PFC which are reflected by their different patterns of connectivity throughout the brain [15]. The orbitofrontal cortex (OFC) is associated with encoding the value of reward [16,17], while the medial PFC including the prelimbic (PrL) and infralimbic (IL) regions are responsible for functions such as decision making, judgements and motor inhibition [18,19,20,21]. Therapeutic doses of MPH alleviate ADHD symptoms through improved performance on tasks that are dependent upon the integrity of the PFC, including those involving working memory and attention [13,22].

Optimal functioning of the PFC requires moderate levels of the catecholamines DA and NA, where either too little or too much of these neurotransmitters results in impaired PFC function [23]. MPH is commonly administered during childhood and adolescence at an age where the PFC is undergoing extensive synaptogenesis and neural remodelling [24]. Furthermore, environmental stimuli and the administration of related psychostimulant drugs can influence synaptic plasticity in the PFC of the rat, and therefore have an ability to alter the function of this region [25,26]. This suggests that the inappropriate administration of MPH may have a pronounced effect on brain regions associated with higher cognition and may alter the development of these regions. 

Most previous pre-clinical research assessing the long-term effects on cognition of chronic treatment with MPH in adolescence has focused on memory performance [27]. However one study has investigated the effect of systemic injections of MPH during adolescence in “normal” rats and found reduced impulsivity during adulthood [28]. The current study aimed to better model oral dosing regimes of MPH in children in order to assess the long-term effect of inappropriate oral administration of MPH on cognition. The drug administration method is important as the dose, time and route of administration all impact the pharmacokinetics of MPH. The effect of this chronic treatment on cognitive function was examined using impulsivity and extinction tasks that are dependent on PFC function [29,30,31]. Using a delayed reinforcement task, impulsive behaviour can be measured as the selection of an immediate small reinforcer over a large reinforcer delivered after a delay [32]. Extinction of this task has also been used as a measure of sustained attention [33,34,35]. In addition to cognitive testing, the effect of chronic MPH administration on the level of tyrosine hydroxylase (TH, the rate limiting enzyme in the synthesis of DA and NA [36]) in PFC subregions was assessed. The administration of MPH at high doses changes the level of TH protein in the PFC of rats [37]. Therefore changes in TH-ir terminal arbours in the PFC could indicate altered signalling from distant catecholaminergic neurons and/or changes to the level of TH availability [38]. 

Appropriate animal models are necessary to assess the long-term effects of MPH treatment on development. The current study used the Wistar-Kyoto rat (WKY) as the “normal” (*i.e.*, misdiagnosed) rat, as it is the genetic control for the Spontaneously Hypertensive/Hyperactive rat (SHR). The SHR has been widely used and extensively studied as an animal model for ADHD [39] and was used as a control strain. The current longitudinal study aimed to investigate the long-term effects on cognitive and neural development of chronic MPH administration during adolescence in the rat. The focus of this study was the impact such treatment has on a misdiagnosed (*i.e.*, WKY) population. To achieve this goal, WKY rats and SHRs were treated with oral MPH throughout adolescence and their performance on cognitive tasks was assessed in adulthood. To measure possible changes to catecholamine function following MPH treatment in WKY rats and SHR, TH-ir was also conducted at different stages of the study: 1 week (short-term group) and 12 weeks (long-term group) following chronic drug treatment, and after cognitive testing (behavioural group, also 12 weeks post treatment).

## 2. Results

### 2.1. Experiment 1—Behavioural Effect of Acute Oral MPH Administration

#### Oral MPH Dose-Response Curves for Locomotor Activity in WKY

**Figure 1:**
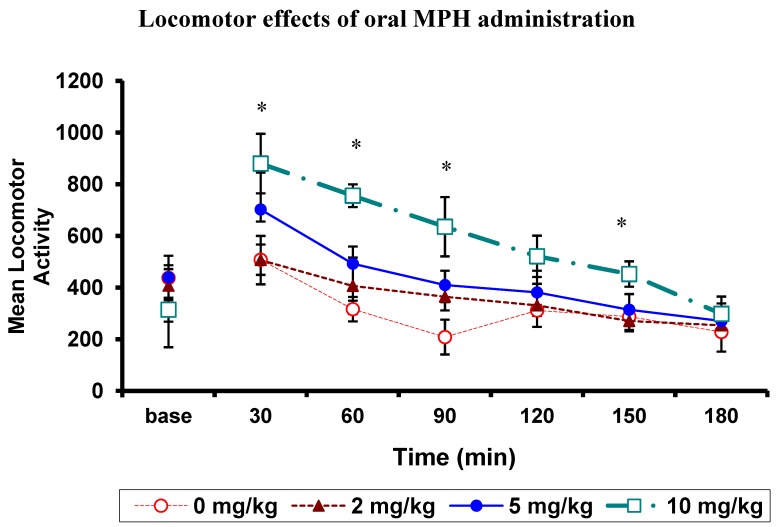
Wistar Kyoto (WKY) rats: Mean locomotor activity (breaks in passive infrared detectors (PIR) beams) at 30 min intervals, prior to (baseline; base) and following oral methylphenidate (MPH) administration over a 3 h period (*n* = 5 per dose). * *p* < 0.017 for a significant difference between the 0 mg/kg and 10 mg/kg doses.

Acute oral MPH administration produced a dose-dependent increase in locomotor activity. As expected there were significant main effects of MPH dose, (*F*(1.352,4.055) = 19.654, *p* = 0.01), and time (*F*(2.071,6.214) = 20.598, *p* = 0.002) on locomotor activity. The locomotor activity over the 3 h was significantly higher following oral administration of the 10 mg/kg dose compared to the 0 mg/kg dose, *p* = 0.001 (Figure 1). Locomotor activity following drug administration reduced over time. The dose by time interaction was also significant, (*F*(2.689,8.066) = 5.051, *p* = 0.032). As illustrated in Figure 1, compared to the 0 mg/kg dose, the 10 mg/kg dose significantly increased locomotor activity in the first, second, third and fifth 30 min intervals, *p*’s = 0.014, 0.001, 0.01, and 0.003, respectively. No significant differences were found between the 0 mg/kg and 2 mg/kg, and the 0 mg/kg and 5 mg/kg doses at any time interval. Baseline locomotor activity was not different between treatment groups.

### 2.2. Cognitive-Behavioural Tasks

#### 2.2.1. Locomotor Activity (During Chronic Treatment: 4 Weeks of 2 × 2 mg/kg Oral MPH or dH_2_O)

##### 2.2.1.1. Locomotor Activity: WKYs

**Figure 2:**
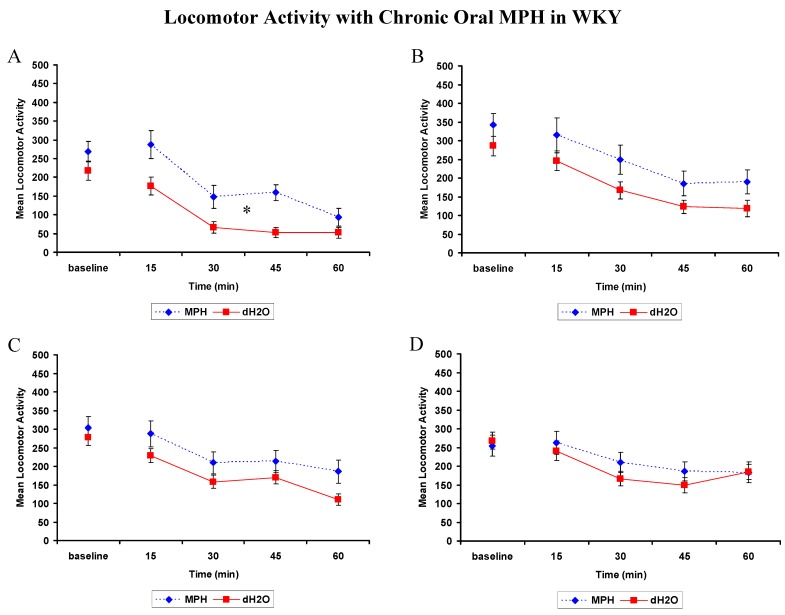
WKY rats: Mean (±SEM) locomotor activity at the beginning of each week of oral treatment with 2 mg/kg methylphenidate (MPH; *n* = 12) or distilled water (dH_2_O; *n* = 12),measured for 15 min intervals prior to (baseline) and for 1 h following treatment. * Significant difference between treatment, *p* < 0.05, in week 1 (**A**) with no significant effect in week 2 (**B**); week 3 (**C**); or week 4 (**D**).

Figure 2 shows baseline locomotor activity in the WKY did not differ between treatment groups across the treatment weeks. Following treatment, there were significant main effects of week (Wilks’ Lambda 0.171, *F* = 32.312, *p* < 0.001), and time (Wilks’ Lambda 0.06, *F* = 74.305, *p* < 0.001). Locomotor activity in both treatment groups was significantly lower in week 1 compared to weeks 2, 3, and 4, *p*’s < 0.05. The week by time by treatment interaction was significant (Wilks’ Lambda is 0.222, *F* = 3.218, *p* = 0.031), with significantly higher locomotor activity in the MPH treated rats in first 45 min following treatment in week 1 only, *p*’s < 0.05, illustrated in Figure 2. There was no main effect of treatment and all other interactions involving treatment were not significant. 

##### 2.2.1.2. Locomotor Activity: SHRs

**Figure 3:**
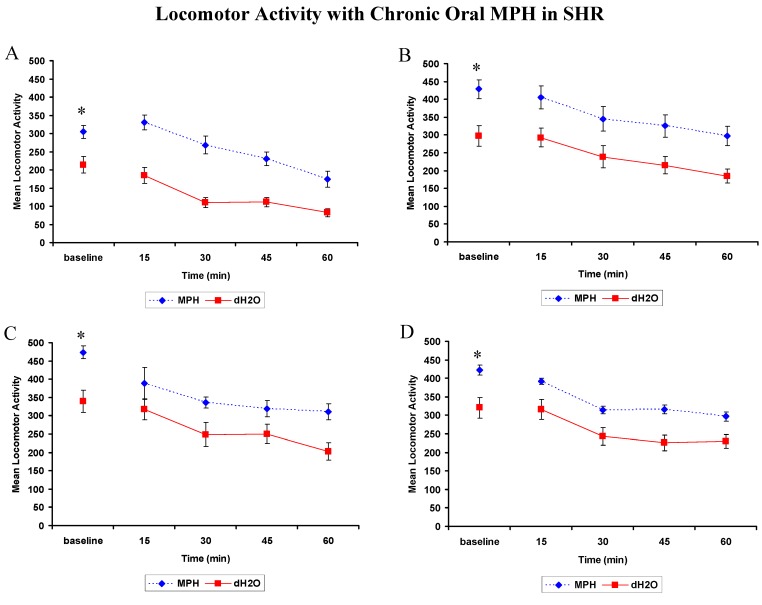
Spontaneously Hypertensive Rats (SHR): Mean (±SEM) locomotor activity at the beginning of each week of oral treatment with 2 mg/kg methylphenidate (MPH; *n* = 9) or distilled water (dH_2_O; *n* = 12), measured for 15 min intervals prior to (baseline) and for 1 h following treatment in week 1 (**A**); week 2 (**B**); week 3 (**C**); and week 4 (**D**). * Significant difference in baseline locomotor activity between treatment groups, *p* < 0.05.

Figure 3 shows baseline activity of the SHR was significantly different between treatment groups for each week (*p*’s < 0.05). Analysis revealed significant main effects of treatment (*F*(1,19) = 14.698, *p* = 0.001), week (*F*(3,57) = 44.33, *p* < 0.001) and time (*F*(4,76) = 103.083, *p* < 0.001). None of the interactions were significant. On average, the MPH treated rats had significantly higher locomotor activity compared to dH_2_O treated rats. Locomotor activity in both treatment groups was significantly lower in week 1 compared to weeks 2, 3, and 4, *p* < 0.05 for all. As illustrated in Figure 3, there was no treatment by time interaction, indicating that the main effect of treatment was attributed to an elevated baseline activity and not a direct result of MPH administration. Therefore MPH and dH_2_O treatment had a similar effect on locomotor activity in the SHRs.

#### 2.2.2. Delayed Reinforcement Task (DRT, 2 Weeks Following 4 Weeks Chronic Treatment)

##### 2.2.2.1. DRT: WKYs

**Figure 4:**
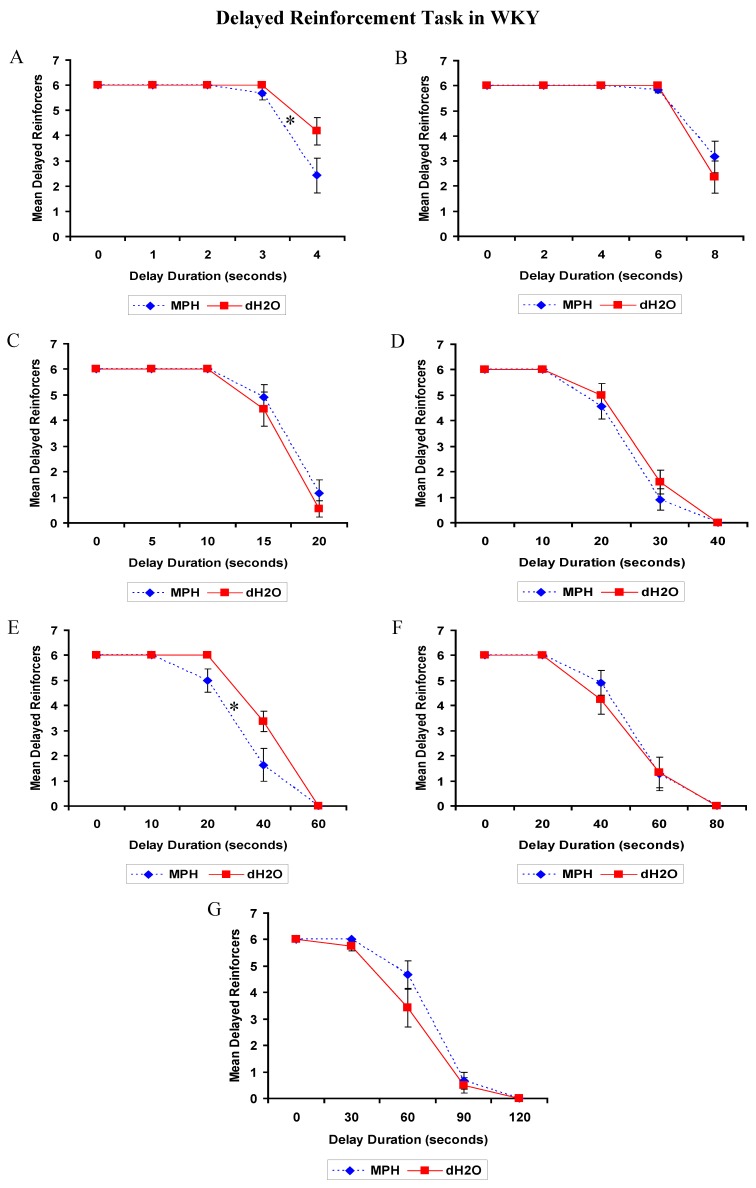
WKY rats: Mean (±SEM) delayed reinforcers attained in Test 1 to 7 (**A–G**) of the Delayed Reinforcement Task by rats previously exposed to 4 weeks of methylphenidate (MPH, 2 × 2 mg/kg/day) or distilled water (dH_2_O) treatment. One test was conducted each day over a period of 7 days (15–21 days post-treatment). * Significant difference between treatments, *p* < 0.05.

There was a significant effect of MPH treatment on delay sensitivity post treatment as measured in test 1 and 5 (see Experimental Section 4.4.1.2 for test parameters), yet not for the remaining tests (Figure 4).

Test 1 (*n* = 12 MPH: *n* = 11 dH_2_O): There were significant effects of treatment (*F*(1,21) = 4.402, *p* = 0.048), and delay (Wilks’ Lambda is 0.351, *F* = 18.491, *p* < 0.001). The treatment by delay interaction was not significant, *p* > 0.05. Rats previously treated with MPH chose the delayed lever significantly less than rats that received dH_2_O pretreatment (Figure 4A).

Test 5 (*n* = 11 per group): There were significant main effects of treatment, (*F*(1,20) = 6.438, *p* = 0.02), and delay, (Wilks’ Lambda is 0.003, *F* = 3421.489, *p* < 0.001). The treatment by delay interaction was not significant, *p* > 0.05. Rats previously treated with MPH choose the delayed lever significantly less than rats that received dH_2_O pretreatment (Figure 4E).

The analyses for Tests 2, 3, 4, 6 and 7 returned similar results to each other. There was a significant effect of delay on each of the tests, *p* < 0.05 for all, such that the longer the delay duration the less the delayed lever was chosen (Figure 4B–D,F,G). The main effects of treatment and the treatment by delay interactions were not significant, *p* > 0.05. WKYs previously treated with MPH were more sensitive to delayed reinforcement in test 1 and 5.

##### 2.2.2.2. DRT: SHRs

**Figure 5:**
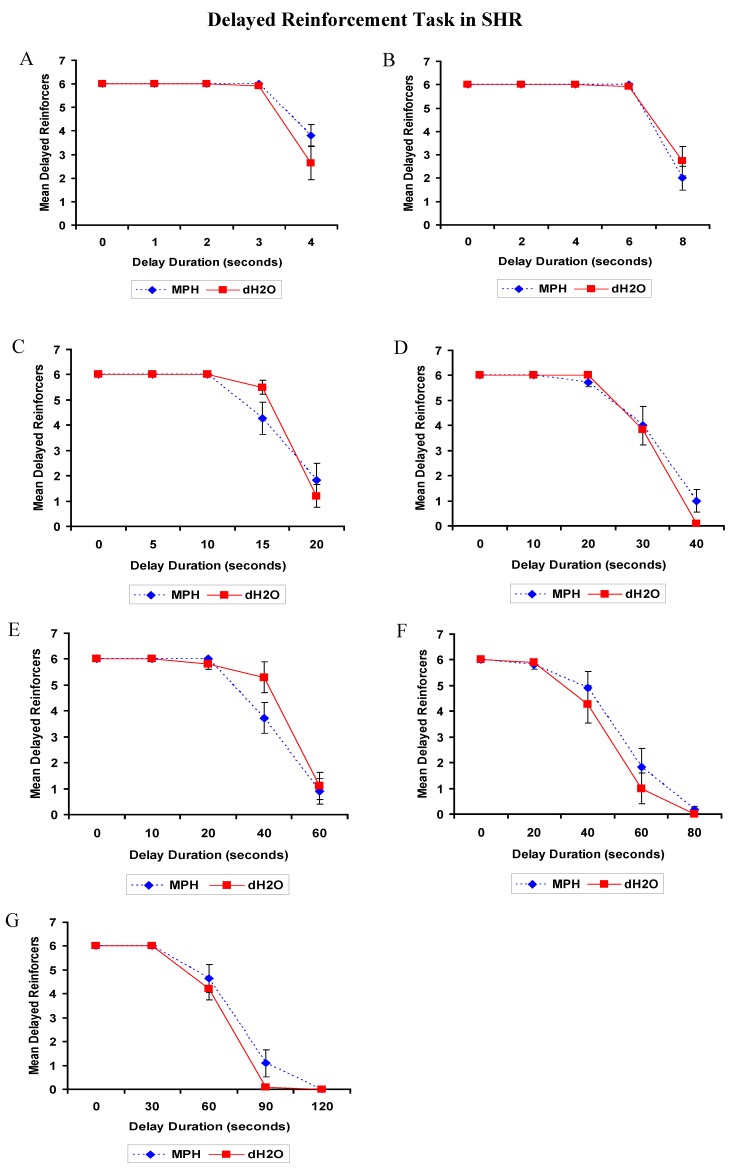
SHR: Mean (±SEM) delayed reinforcers attained in Test 1 to 7 (**A–G**) of the Delayed Reinforcement Task by rats previously exposed to 4 weeks of methylphenidate (MPH, 2 × 2 mg/kg/day) or distilled water (dH_2_O) treatment. One test was conducted each day over a period of 7 days (15–21 days post-treatment).

The analyses for all tests (1 to 7) returned similar results. There was a significant effect of delay on each of the tests, *p* < 0.05 for all, such that the longer the delay duration the fewer times the delayed lever was chosen. The main effects of treatment and the treatment by delay interactions were not significant, *p*’s > 0.05. There was not a significant effect of prior exposure to chronic MPH treatment on impulsivity in SHRs (Figure 5). 

#### 2.2.3. Extinction Task (EXT, 3 Weeks Following Chronic Treatment)

##### 2.2.3.1. EXT: WKYs

**Figure 6:**
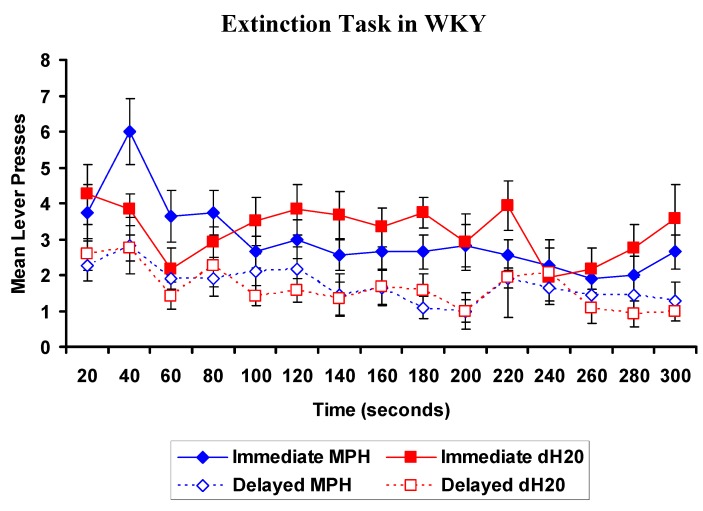
WKY rats: Mean (±SEM) number of presses on the immediate (closed) and delayed (open) levers in consecutive 20 s intervals for rats previously exposed to 4 weeks of oral methylphenidate (MPH, 2 × 2 mg/kg/day; blue diamond; *n* = 11) or distilled water (dH_2_O; red squares; *n* = 12), during the extinction task. There was a significant lever by time by treatment interaction, *p* = 0.039. The extinction task was conducted 48 h following the final DRT test.

At the commencement of the EXT task, WKYs previously treated with MPH pressed the immediate lever more than WKYs previously treated with dH_2_O. There was a significant main effect of lever, (Wilks’ Lambda is 0.276, *F* = 55.173, *p* < 0.001) such that WKYs pressed the immediate more than the delayed lever in the EXT task (Figure 6). There was a significant lever by time by treatment interaction (Wilks’ Lambda is 0.139, *F* = 3.540, *p* = 0.039), indicating that MPH pretreatment increased immediate lever pressing. There was no main effect of treatment and all other interactions were not significant. MPH treated WKYs continued to demonstrate sensitivity to delay. 

##### 2.2.3.2. EXT: SHRs

**Figure 7:**
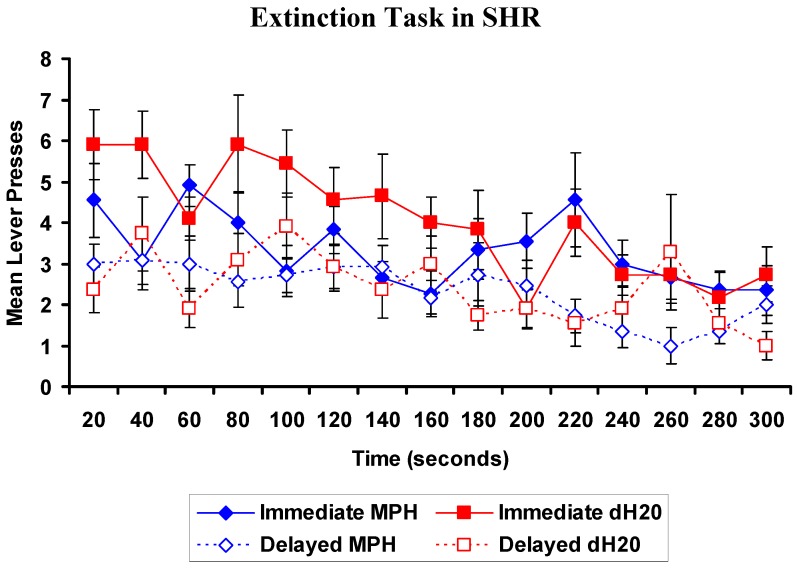
SHR: Mean (±SEM) number of presses on the immediate (closed) and delayed (open) levers in consecutive 20 s intervals for rats previously exposed to 4 weeks of oral methylphenidate (MPH, 2 × 2 mg/kg/day; blue diamond; *n* = 11) or distilled water (dH_2_O; red squares; *n* = 11), during the extinction task. The extinction task was conducted 48 h following the final delayed reinforcement tasks (DRT) test.

Analysis found a significant main effect of lever, Wilks’ Lambda is 0.491, *F* = 20.701, *p* < 0.001, and a significant main effect of time, Wilks’ Lambda is 0.123, *F* = 3.556, *p* = 0.049 (Figure 7). SHRs pressed the immediate lever more than the delayed lever and as time progressed lever pressing decreased. There was no main effect of treatment and all interactions involving treatment were not significant, *p* > 0.05. MPH treatment of SHRs did not affect lever pressing during the EXT task compared to dH_2_O treated controls.

### 2.3. TH Immunoreactivity in PFC

The current study measured the levels of TH-ir in the PFC at different times (1 and 12 weeks post chronic drug treatment, short-term and long-term groups, respectively) and following cognitive testing (behavioural group, also 12 weeks post treatment) to assess possible changes to catecholamine function following MPH treatment in WKY rats and SHRs.

#### 2.3.1. Results for WKYs

**Figure 8:**
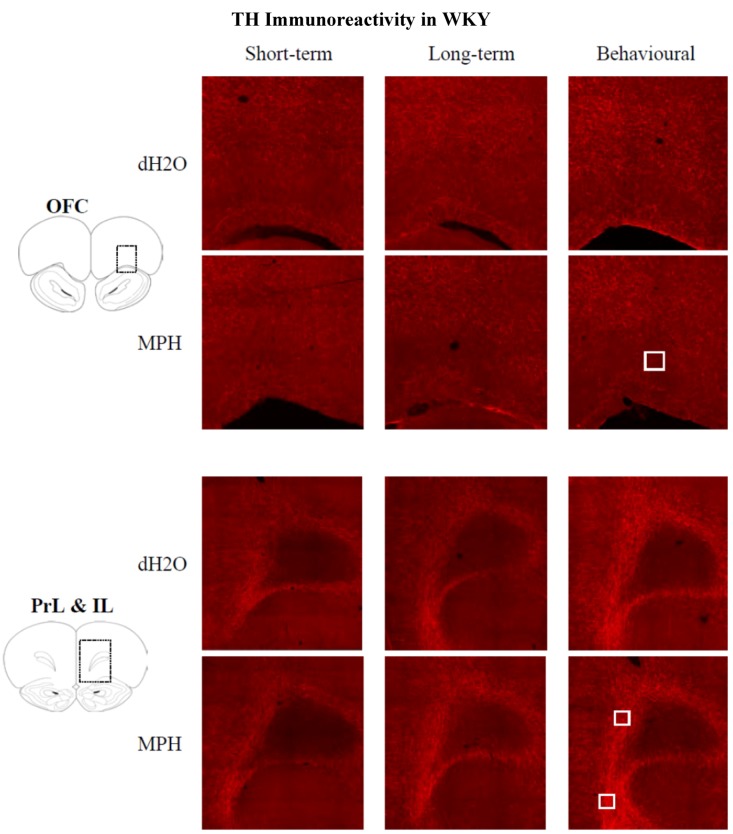
WKY rats: Images of tyrosine hydroxylase-ir fibres in the orbitofrontal cortex (OFC), prelimbic cortex (PrL; dorsal) and infralimbic cortex (IL) following chronic treatment with either methylphenidate (MPH) or distilled water (dH_2_O). Immunohistochemical staining was conducted in the short- and long-term groups, 1 and 12 weeks after cessation of treatment, respectively, and following behavioural testing (12 weeks post treatment) in the behavioural group. The white square represents the analysis probe in each region of interest (50,000 µm^2^). Images have been adjusted for presentation.

Immunohistochemistry identified TH-ir fibres throughout all regions of the PFC with a high density of labelling in layers 5 and 6 of the prelimbic cortex (PrL) and infralimbic cortex (IL) (Figure 8). 

**Figure 9:**
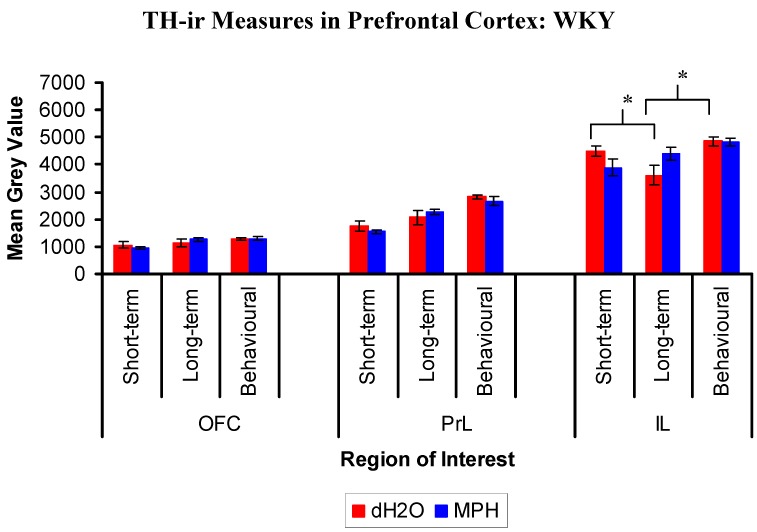
Mean (±SEM) tyrosine hydroxylase immunoreactivity in the lateral orbiotofrontal cortex (OFC), the prelimbic cortex (PrL), and infralimbic cortex (IL) of WKY rats, previously exposed to chronic methylphenidate (MPH, 2 × 2 mg/kg/day) or distilled water (dH_2_O) treatment. Immunohistochemical staining was conducted in the short- and long-termgroups, 1 and 12 weeks after cessation of treatment, respectively, and following behavioural testing (12 weeks post treatment) in the behavioural group. Within each region of interest, there was a significant main effect of group such that the behavioural group had significantly more TH staining compared to the short-term and long-term groups, averaged across treatment. * Significant difference for the comparisons of interest for dH_2_O treated WKYs between the short- and long-term groups, and the long-term and behavioural groups, *p* < 0.05.

In orbitofrontal cortex (OFC), analysis of the treatment and group differences in the mean grey value revealed a main effect of group, (*F*(2,18) = 5.28, *p* = 0.016 (Figure 9)). This effect was such that the short-term group had significantly less TH staining compared to the long-term (*p* = 0.035) and behavioural (*p* = 0.006) groups, averaged across treatment. There was no main effect of treatment, (*F*(1,18) = 0.006, *p* = 0.937), and the treatment by group interaction was not significant, (*F*(2,18) = 1.028, *p* = 0.378).

In the PrL analysis of the mean grey value there was a main effect of group, (*F*(2,18) = 23.508, *p* < 0.001 (Figure 9)). This effect was such that the behavioural group had significantly more TH staining compared to both the short-term (*p* < 0.001) and long-term (*p* = 0.002) groups, averaged across treatment. There was no main effect of treatment, (*F*(1,18) = 0.249, *p* = 0.624), and the treatment by group interaction was not significant, (*F*(2,18) = 0.938, *p* = 0.41).

In the IL analysis of the treatment and group differences in the mean grey value there was a main effect of group, (*F*(2,18) = 6.259, *p* = 0.009 (Figure 9)). This effect was such that the behavioural group had significantly more TH staining compared to the long-term (*p* = 0.003) and short-term (*p* = 0.017) groups, averaged across treatment. There was no main effect of treatment, (*F*(1,18) = 0.068, *p* = 0.797). As seen in Figure 9, the treatment by group interaction was significant, (*F*(2,18) = 3.853, *p* = 0.04), indicating that the group differences were significantly changed following treatment. In WKYs treated with dH_2_O, there was significantly less TH staining in the long-term compared to the short-term group (*p* = 0.039), while the behavioural group had significantly more TH staining compared to the long-term group (*p* = 0.007). In WKYs treated with MPH, no group differences were evident (*p*’s > 0.05). Thus, MPH treatment has altered the control pattern of TH positive fibres across the short-term, long-term and behavioural groups. 

#### 2.3.2. Results for SHRs

**Figure 10:**
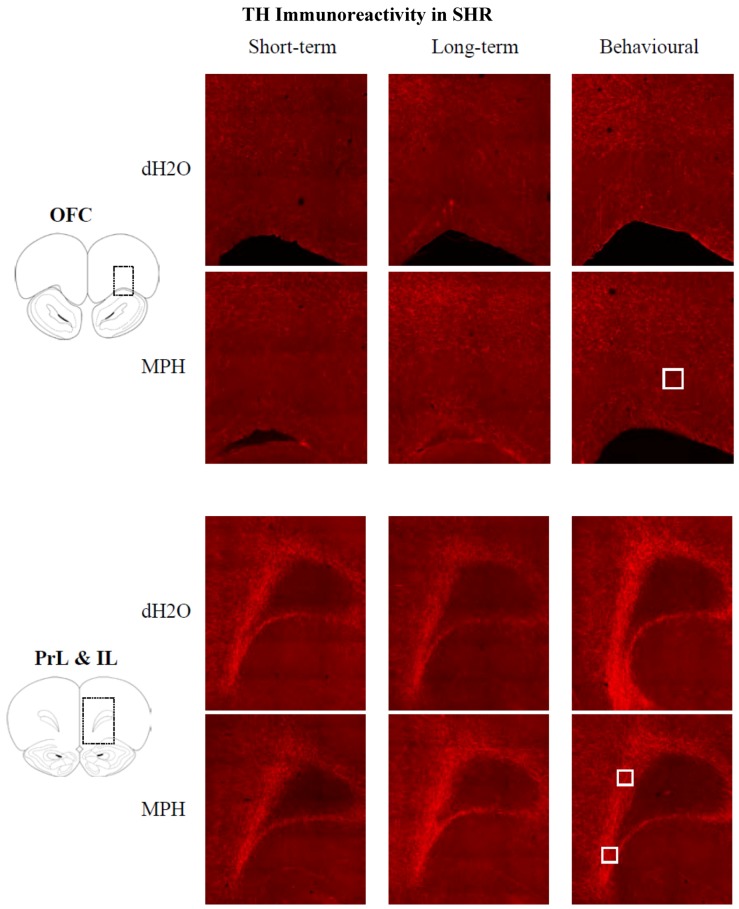
SHR: Images showing tyrosine hydroxylase-ir fibres in the orbitofrontal cortex (OFC), prelimbic cortex (PrL; dorsal) and infralimbic cortex (IL) following chronic treatment with either methylphenidate (MPH) or distilled water (dH_2_O). Immunohistochemical staining was conducted in the short- and long-term groups, 1 and 12 weeks after cessation of treatment, respectively, and following behavioural testing (12 weeks post treatment) in the behavioural group. The white square represents the analysis probe in each region of interest (50,000 µm^2^). Images have been adjusted for presentation.

TH-ir fibres were seen throughout all regions of the PFC with a high density of TH staining in layers 5 and 6 of the PrL and IL (Figure 10). 

**Figure 11:**
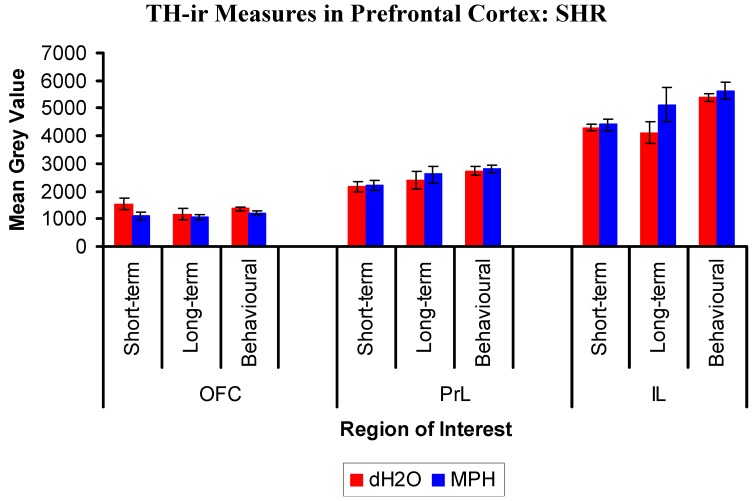
Mean (±SEM) tyrosine hydroxylase-ir in the lateral orbitofrontal cortex (OFC), the prelimbic cortex (PrL), and infralimbic cortex (IL) of SHRs, previously exposed to chronic methylphenidate (MPH, 2 × 2 mg/kg/day) or distilled water (dH_2_O) treatment. Immunohistochemical staining was conducted in the short- and long-term groups, 1 and 12 weeks after cessation of treatment, respectively, and following behavioural testing (12 weeks post treatment) in the behavioural group. Within the IL region, there was a significant main effect of group such that the behavioural group had significantly more TH staining compared to the short-term and long-term groups, averaged across treatment.

The OFC analysis of the treatment and group differences in the mean grey value showed no main effect of group, (*F*(2,18) = 1.21, *p* = 0.321), or treatment, (*F*(1,18) = 3.628, *p* = 0.073 (Figure 11)). The treatment by group interaction was not significant, (*F*(2,18) = 0.796, *p* = 0.466).

The PrL analysis of the treatment and groups differences in the mean grey value showed no main effect of group, (*F*(2,18) = 3.326, *p* = 0.059), or treatment, (*F*(1,18) = 0.379, *p* = 0.546 (Figure 11)). The treatment by group interaction was not significant, (*F*(2,18) = 0.061, *p* = 0.941).

The IL analysis of the treatment and group differences in the mean grey value revealed a main effect of group, (*F*(2,18) = 5.995, *p* = 0.01 (Figure 11)). This effect was such that the behavioural group had significantly more TH staining compared to the short-term (*p* = 0.004) and long-term (*p* = 0.021) groups, averaged across treatment. There was no main effect of treatment, (*F*(1,18) = 2.644, *p* = 0.121), and the treatment by group interaction was not significant, (*F*(2,18) = 0.947, *p* = 0.407).

## 3. Discussion

The present study sought to identify enduring cognitive effects produced by chronic MPH treatment during development in “normal” WKY subjects. Methylphenidate was chronically administered to adolescent rats (PND 27-52) using a novel oral administration method with a clinically relevant dosing regime. The findings that there was an increase in impulsive choice and sensitivity to delay in adulthood, and alteration to the regular progression of staining for TH in the IL cortex, suggest that chronic MPH treatment to WKY rats, resulted in altered cognitive performance in adulthood. In contrast to the WKY, chronic MPH treatment during adolescence in SHRs had no long-term effects on cognition or catecholamine neural development at adulthood. 

These findings are consistent with previous research reporting strain differences in acute behavioural and locomotor effects of MPH [39,40] and *d*-amphetamine [41]. It was hypothesised that MPH treatment would reduce SHR locomotor activity as previous research has demonstrated attenuated hyperactivity in the SHR following psychostimulant treatment [42]. The dose of MPH employed in this study was determined by the initial oral dose-response curve, in conjunction with previous research assessing blood plasma levels following oral administration of MPH in Sprague-Dawley rats [13,43]. Our study demonstrated, in WKYs, that the novel oral administration of MPH produced similar dose-dependent increases in locomotor behaviour to Sprague-Dawley rats treated with MPH via gavage [44]. Given the differences in MPH effect between WKYs and SHRs [39,40,45], it is possible that the dose used in this study was not therapeutically relevant for the SHRs. It should also be noted that the presence of hyperactivity in this strain is age-dependent [46] and the variability in baseline locomotor activity between the two treatment groups may highlight the different levels of development in this strain. However, regardless of baseline activity levels, acute or chronic MPH administration did not alter locomotor activity in the SHR in our study.

With respect to chronic MPH treatment, the locomotor results for the WKYs were not entirely as expected. Based on previous research [13,44], the therapeutic dose of MPH administered in the current study should not affect locomotor activity in the WKY. The acute increase (week 1) in locomotor activity in the chronic study is also not consistent with the results of our acute locomotor dose-response curve, which found no locomotor activation following the oral administration of 2 mg/kg dose of MPH. This inconsistency could have occurred due to age difference (2 weeks) between the groups at the time of initial drug administration as age has been shown to play a significant role in the effect of MPH [47]. From the second week of treatment there was no increase in activity with MPH administration. It might also be expected that chronic treatment with this psychostimulant would produce enhanced or “sensitized” locomotor activity [48], however the lack of sensitized response in the current study is consistent with previous research reporting no increase in locomotor activity following acute *therapeutic* doses of MPH in “normal” rats [13,44]. Similar findings have also been reported following amphetamine dosing at a therapeutic level [49]. The current study is the first to monitor locomotor activity in adolescent rats throughout chronic therapeutically relevant administration of MPH. 

It was hypothesised that WKYs treated with MPH would demonstrate long-term changes in cognitive performance. Following treatment, MPH pretreated WKYs were found to be more impulsive and sensitive to delays compared to WKYs pretreated with dH_2_O. This study is the first to report increased impulsive choice following inappropriate, chronic MPH treatment in rodents. The findings imply that WKYs chronically treated with MPH throughout adolescence are more susceptible to the influence of immediate gratification where their behaviour is not as strongly guided by the long-term consequences of their actions. However, the results of this task are unable to determine whether the previous chronic MPH treatment produced an actual altered sensitivity to the increasing delay or the value of the reward. The findings of the extinction task suggest that the elevated impulsivity of the WKYs chronically treated with MPH throughout adolescence may result from an altered sensitivity to the increasing delay as they initially chose the immediate lever more than the delayed lever. As the rats were drug free when impulsivity testing was conducted, it is unlikely that the appetite suppressive effects of MPH could explain the altered response pattern of WKYs previously treated with MPH.

Our findings are not consistent with those of Adriani and colleagues [28] who reported a reduction of impulsivity in “normal” adult rats pretreated with MPH. The inconsistent findings are likely due to different methods of MPH administration. The current study delivered a low (2 mg/kg), oral dose of MPH twice a day during the rats’ active period in their circadian cycle, while the Adriani study administered a single daily dose (2 mg/kg), via i.p. injection during the light period or “inactive” phase for the rats. The latter method is not consistent with clinical dosing regimes in which children are orally treated with low doses of MPH, during the “active” phase of their circadian cycle [43]. Furthermore, an i.p. injection of 2 mg/kg MPH would result in rapid elevation and higher peak plasma concentrations of the drug than are considered therapeutically relevant [44,50]. Using a method of drug administration that more closely reflects the dosing regime employed clinically, the results of the current study suggest that inappropriate treatment with MPH may increase aspects of impulsivity in adulthood.

The MPH induced impulsivity in adulthood is consistent with previous reports of PFC dysfunction following chronic psychostimulant drug administration. Cocaine is a psychostimulant with a similar mechanism of action to MPH [10,12]. In rats, the PFC mediated task of reversal learning is impaired following chronic treatment with high dose cocaine, with similar impairments occurring in monkeys across lower dose ranges [51,52]. As reversal learning involves the inhibition of a previously rewarded response, impulsive behaviour has been associated with impairments of reversal learning [53]. The results from the current study expand and extend the previous findings by demonstrating similar long-term changes in impulsive-like behaviour following prior chronic treatment with low doses of the psychostimulant MPH.

Our results show that chronic MPH administration produced long-term effects on cognitive performance in WKY rats but not in the SHRs. It is thought that the therapeutic action of low doses of MPH in ADHD is to increase abnormally low levels of DA and NA in the PFC, increasing neural activity and improving cognitive performance [14,22]. The administration of MPH to those with “normal” catecholamine function also alters cognitive function, with low doses enhancing performance [13] and higher doses increasing catecholamine levels in excess of what is optimal, resulting in PFC impairment [23,54]. The long-term behavioural effects of chronic MPH treatment in the present study may be consistent with the hypothesis that psychostimulants produce a persistent reorganisation of patterns of synaptic connectivity in brain regions including the PFC, which may impair cognitive behaviour in “normal” WKY rats [25]. 

The current study measured the levels of TH in the PFC at different times (1 and 12 weeks post chronic drug treatment, short-term and long-term, respectively) and following different experiences (behavioural testing, 12 weeks post drug treatment) to assess possible changes to catecholamine function following MPH treatment. The influence of MPH to prevent the pattern of progressive change of TH staining across the groups in the IL region of the WKY rats, but not the SHRs, suggests that MPH administration may interfere with brain maturation and response to experience in the WKY strain. This finding is in line with enhanced impulsivity in WKYs pretreated with MPH as the IL region is involved with impulsive actions, an aspect of impulsivity [55]. A comparison of TH protein levels between the short-term and long-term groups determined the influence of time on catecholamine neuron development. For the dH_2_O treated WKYs, this comparison revealed that the TH density of the older rats (long-term) was reduced compared to the younger rats (short-term) in all areas measured in the PFC. Similar patterns of changes in TH protein over the development of the PFC have been shown in the rhesus monkey [56]. Rosenberg and Lewis [56] reported a rapid rise in TH positive fibres during infancy and early childhood, followed by a decline from 2 to 3 years of age until reaching a stable level in adulthood. These findings are also consistent with human and animal research that demonstrates pruning or reduction of superfluous synapses and neurons that occurs in the frontal cortices until early adulthood [57,58]. As there was no such reduction in the TH staining in the IL for the WKYs treated with MPH in the long-term group, this suggests that MPH treatment during adolescence may interfere with the catecholaminergic maturation of the PFC.

The comparison between the WKYs in the long-term and behavioural groups shows alterations in TH density due to cognitive-behavioural testing. Such cognitive-behavioural tests are considered a component of behavioural environmental enrichment [59] and enriching experiences have been shown to alter brain morphology [26]. The increased level of TH density in the IL of the dH_2_O treated WKYs in the behavioural group suggests increased neural complexity following behavioural enrichment. Notably, this difference between the long-term and behavioural groups was not evident for WKYs treated with MPH, suggesting that treatment with psychostimulants may limit appropriate neuroadaptations [60]. However, this limiting effect of chronic MPH treatment on TH fibres does not agree with previous research by Gray and colleagues [36] that reported an increase of TH density in the PFC following the last dose of 28 days of chronic MPH treatment. This likely reflects a high dose i.p. administration of MPH and the timing of TH measures. Gray *et al.* [36] measured elevated TH density on the last day of their treatment regime, while the present study employed a minimum delay of 1 week between cessation of treatment and tissue analysis. It is usual that experimental measures are collected at least a week following cessation of treatment to avoid measures of transient changes brought about by withdrawal symptoms [61]. In the present study, it is likely that the rats had progressed past the withdrawal phase and were exhibiting enduring changes in impulsive choice as the behavioural measures were attained over two weeks after the final dose of MPH. Similar periods between discontinuation of chronic psychostimulant treatment and assessment of enduring changes have been employed in previous studies [62,63]. These current results suggest that MPH treatment in the WKYs, but not the SHRs, interferes with the catecholamine development that would normally occur following aging and exposure to enriching environments. Similar strain differences in TH activity following reserpine treatment have also been reported [64].

A significant consideration for the current study is the validity of the WKY as a “normal” rat strain [65,66,67]. However, as we have previously shown in this laboratory [68], WKYs were not found to be hypoactive compared to SHRs in operant tasks. Furthermore, the behavioural stimulation following the high oral dose of MPH administration to WKYs in the present study was consistent with increased locomotor activity observed in Sprague-Dawley rats following equivalent dosing [44]. It would be prudent to include additional “normal” rat strains to establish that reported group differences are not the result of deficits in the WKY. However, consideration must also be given to the large genetic variation that would be introduced by including such out-bred strains.

The current findings have important implications for children chronically treated with MPH. If children have been misdiagnosed with ADHD and are inappropriately treated with MPH, these findings suggest that the chronic treatment may induce impulsive behaviours in adulthood. Further investigation of the neural mechanisms underlying persistent cognitive changes following chronic MPH treatment is necessary. MPH is commonly prescribed to children and adolescents to treat ADHD, with prescriptions rates rapidly rising in the last decade [69,70,71,72]. This emphasizes the need for more clinically appropriate research to elucidate the long-term effects of MPH treatment. It is imperative that future research employs dosing regimes that allow the results to be applicable to the human situation. Furthermore, future research would benefit from investigating the effects of chronic MPH treatment at different ages, early, mid, late adolescence, and even adulthood to determine if one period of development is more sensitive to pharmacological intervention than another. This research not only has implications for children misdiagnosed with ADHD but also for those who deliberately misuse MPH.

## 4. Experimental Section

### 4.1. Subjects

Forty-five male WKY and 40 male SHR rats were obtained from the Animal Resources Centre (Canning Vale, WA, Australia). One SHR was not well and was therefore excluded from the study. Upon arrival in the laboratory, the rats were housed individually in opaque, plastic cages (60 × 21.5 × 36 cm, length × height × width) containing sawdust, a block of wood and shredded paper. The cage was covered with a raised wire mesh roof (27 cm total height). The animal holding room was held at a constant temperature of 21 ± 1 °C. Rats were housed on a reverse light/dark cycle (lights on at 20:00 p.m. until 08:00 a.m.) and experiments were conducted during the rats’ active (dark) cycle. At the beginning of the procedure, the rats were approximately 25 days old, weighed 51–85 g (WKY) and 46–90 g (SHR), had been handled daily for one week by the experimenter and were experimentally naïve. They were allowed free access to water and standard laboratory rat chow, except during the drug administration and cognitive assessment procedures as detailed in the relevant sections below. The rats were individually housed to facilitate drug administration with minimal handling of the animals and to eliminate competition by littermates for food and water during these periods of restriction. Rats were weighed daily to determine treatment volume (1 mL/kg) and to monitor growth. 

The study was conducted with the approval of the Macquarie University Animal Ethics Committee (reference number ARA 2006/019) and followed the Australian Code of Practice for the Care and Use of Animals for Scientific Purposes [73]. 

### 4.2. Drug Administration Procedure

Ritalin tablets (10 mg/tablet, Novartis, East Hanover, New Jersey) were crushed and suspended in distilled water (dH_2_O, 1 mg/mL) and administered through a drinking spout. The drinking spout was a metal tube inserted into a rubber stopper, which had a ball-bearing at the end to hold the water in the tube. The drinking spout was assessed for leakage prior to and following drug administration.

Rats were placed on water restriction in order to facilitate drug administration via the drinking spout. The drinking spout was placed in the cage and 1 mL of water was inserted. Only when the rat began drinking was the drug dose inserted into the spout via a syringe (based on body weight (1 mL/kg), typically 0.1–0.3 of 1 mL). Once the drug was added to the spout, an additional 1 mL of water was inserted into the spout to ensure the entire dose had been consumed. This method of drug delivery was designed for oral drug administration, which did not require extended periods of training by gavage. It was observed that the rats voluntarily drank the MPH suspension through a drinking spout following water restriction. The rats consumed all of the liquid administered through the spout in approximately 30 s.

### 4.3. Experiment 1: Acute Drug Dose-Response Curve for Locomotor Activity

Five WKY rats were used to determine the oral dose of MPH for use during chronic treatment. Rats were placed on water restriction for 23 h before drug administration and locomotor testing. Prior to administration of the drug, the rats’ baseline locomotor activity was measured in an operant chamber for 30 min (for apparatus see 3.4.1.1). A latin square design was used to assign each of the four doses (0 mg/kg, 2 mg/kg, 5 mg/kg, 10 mg/kg) to each rat, separated by at least 48 h. Following oral drug administration, the rats were given 5 min *ad libitum* access to water and returned to the locomotor chambers to measure their activity over the following 3 h. Upon completion of the session, the rats were returned to their home cage and given one hour *ad libitum* access to water.

On the days between treatments, the rats were restricted to one hour *ad libitum* access to water to facilitate the administration of the drug on the following day. Once all locomotor sessions had been conducted for each of the test doses, the rats were taken off water restriction and given *ad libitum* access to water.

### 4.4. Experiment 2: General Chronic Treatment Procedure

All groups underwent 4 weeks of either chronic oral MPH or dH_2_O administration during adolescence (postnatal day 27–52) [74]. Following chronic treatment, 3 main groups were formed for immunohistochemical analysis of the PFC; (1) a short-term group (8 WKY, 8 SHR); (2) a long-term group (8 WKY, 8 SHR); and (3) a behavioural group. The short-term and long-term groups were euthanized at 1 week and 12 weeks, respectively, after cessation of chronic treatment to allow assessment of changes to TH immunostaining at times reflecting the beginning and end of the behavioural tasks. The behavioural group (24 WKY, 23 SHR) were euthanized upon completion of cognitive-behavioural testing for neural tissue analysis (12 weeks post treatment). With the exception of performing the cognitive-behavioural tests, rats in the long-term and behavioural groups had the same experiences. The long-term group rats experienced identical levels of food and water restriction during the 12 weeks following treatment as did rats in the behavioural group.

After an initial 23 h of water restriction, rats were familiarized to the dosing procedure by exposure to a sham dose of water through the drinking spout. The following day treatment commenced using this procedure. Rats were chronically treated with either Ritalin (2 mg/kg oral; MPH) or distilled water (dH_2_O) to model clinical dosing in children. Doses were based on the findings of Kuczenski and Segal [43], Berridge *et al.* [13], and the results of the dose-response curve of 3.3.

The Ritalin^®^ suspension was administered through the drinking spout twice a day, five days per week for four weeks. Drug free weekends were employed in the current study as “weekend holidays” are used in the clinical setting and have been shown to reduce the incidence of side-effects [75]. On the first week of treatment, the initial drug administration was given 23 h after the sham dose during which time the rats were water restricted. The first drug administration for each of the following weeks was given after 23 h of water restriction at the end of their rest days. Immediately following consumption of the liquid in the drinking spout from the first daily dose, the rats were given five minutes *ad libitum* access to water. The second daily drug administration was identical and occurred five hours after the first dose; however the rats were allowed one hour *ad libitum* access to water following the second dose. After the second drug administration on the fifth day of the week, the rats were given *ad libitum* access to water for approximately 40 h, at which time they were placed back on water restriction to facilitate drug administration in the following week. The schedule of water restriction was based on previous research allowing rats *ad libitum* access to water on rest days [7,76]. Once the rats had completed four weeks of treatment, they were taken off water restriction and allowed *ad libitum* access to food and water for one week until the cognitive testing commenced. 

#### 4.4.1. Cognitive-Behavioural Tasks

Twenty-four WKY rats and 23 SHR rats completed a number of cognitive-behavioural tasks. Locomotor activity was measured at the beginning of each week of treatment. One week following chronic treatment, cognitive tasks that were completed were a delayed reinforcement and extinction tasks, followed by a radial arm maze (RAM) task. Training for the DRT was conducted 7–14 days post-treatment and all tests for the DRT were conducted over a period of 7 days (15–21 days post-treatment). The extinction task was performed at 48 h after the last DRT test. For brevity, the data for the RAM has been excluded as no effects of treatment were observed in either strain. Brains were collected for TH immunostaining following these tasks at 12 weeks post chronic treatment.

##### 4.4.1.1. Locomotor Activity

Locomotor activity was used as an initial index of pharmacological activation by oral MPH in experiment 1. For experiment 2, locomotor activity was used as a simple method of measuring behavioural change throughout repeated MPH administration. A more detailed description of the apparatus and procedure used to measure locomotor activity is available in Pardey *et al.* [68]. Briefly, the rats were placed in operant conditioning chambers (purpose built by the University of Sydney, Australia) with two passive infrared detectors (PIR, Quantum passive infrared motion sensor, Ness Security Products, Australia) located opposite each other, 30 mm above the floor. Locomotor activity was measured by detection of small movements of the subjects’ head and body. These movements were tracked via “Workbench Mac” software running on Macintosh Computers for one hour [77]. Cameras in each chamber allowed observation of the rats to monitor their welfare during the session. To habituate the rats to the chambers, they were placed in the chambers for 1 h on the day prior to the initial data collection day. Locomotor activity of the rats was measured for 15 min prior to (weekly baseline measure) and 1 h immediately following their morning dose on the first day of each week of treatment. 

##### 4.4.1.2. Delayed Reinforcement (DR) and Extinction (EXT) Tasks

The delayed reinforcement (DR) and extinction (EXT) tasks have been described in more detail in Pardey *et al.* [68]. The animals were placed on food restriction 24 h prior to the commencement of the DR task to maintain their body weight at approximately 85%. There was *ad libitum* access to water in their home cage throughout the DR and EXT tasks. The food reinforcer used was 45 mg Noyes Precision Pellets, Formula A (Research Diets, Inc., New Brunswick, NJ, USA). For both tasks the rats were placed in operant conditioning chambers in which the test wall contained two cue lights, above two levers, on either side of a food magazine.

In the DR task, the rats were trained to press one lever to receive a small reinforcer (one pellet) immediately and the other lever to receive a large reinforcer (five food pellets) after a two second delay. To reduce the risk of partial reinforcement, during the delay the cue light above the activated lever flashed (0.6 s per on/off cycle). Once the animals had been trained, *i.e.*, they pressed each lever 15 times on three consecutive days, they progressed to the test phase. If a rat had not met this criterion within 7 days they were removed from the task. 

For each test, the rats were allowed to choose which lever they pressed and therefore the size of reinforcer they would receive. The reinforcer associated with both levers remained constant throughout the test phase. If the rat chose the immediate lever, they received one pellet immediately. If the rats chose the delayed lever, they received five pellets; however the delay between their response and reinforcer delivery increased during each test session, with increasing delay durations on each of the tests as shown in Table 1. A delay duration was experienced six times before proceeding to the longer delay on that particular test. Delay durations were based on Evenden and Ryan [32] and Adriani and Laviola [78]. As with the training phase, during the delay the cue light flashed until the reinforcer was delivered. The longest delay accepted for each test, the total number of reinforcers attained from each lever and the total number of presses on each lever was recorded.

**Table 1:** Duration of delay (seconds) before reinforcer was delivered on each test.

Test	Delay duration in seconds (each experienced 6 times)
1	0, 1, 2, 3, 4
2	0, 2, 4, 6, 8
3	0, 5, 10, 15, 20
4	0, 10, 20, 30, 40
5	0, 10, 20, 40, 60
6	0, 20, 40, 60, 80
7	0, 30, 60, 90, 120

Following Test 7, all animals were placed on a constant 60 s delay schedule for one day prior to the EXT task. During the EXT task the animals were placed in the operant conditioning chambers as per tests in the DR task, however no food reinforcers were delivered. Lever pressing had no effect on the delivery of a reinforcer or the illumination of the cue lights. Lever pressing was recorded at 20 s intervals over the 5 min duration of the EXT task. At the end of the EXT task all rats were taken off food restriction and had *ad libitum* access to food and water for nine days until water restriction commenced for the RAM.

### 4.5. Immunohistochemistry

#### 4.5.1. Procedure

Rats were deeply anesthetized with lethobarb (pentobarbitone sodium, 80 mg/mL) and received an intra-cardiac injection of 0.5% sodium nitrate and 200 U heparin in saline. Rats were then perfused transcardially with 300 mL of phosphate buffered saline (PBS) followed by fixation with 400 mL of 4% paraformaldehyde/0.1 M PB. Brains were removed and stored in the same fixative at 4 °C overnight. The following day, the brains were coronally blocked at the level of the hypothalamus and returned to cold fixative for a minimum of 3 h. Thereafter, the tissue block was immersed in a graded series of sucrose solutions (15% and 30% in PBS) at 4 °C until the tissue sank to the bottom of the container. The brains were then transferred to cryoprotectant (30% RNase-free sucrose, 30% ethelyene glycol, 1% polyvinylpyrrolidone (PVP-40) in 0.1 M sodium phosphate buffer, (pH 7.4). and stored at −20 °C until processed for TH immunohistochemistry.

The rostral portion of the brain was removed from cryoprotectant and washed twice for 10 min in PBS with 0.1% Tween 20 (PBT). The tissue was sectioned in a 1:4 sequential series into 50 µm coronal slices using a vibrating microtome (Leica 1000S). One series from each brain was reacted, with the other 3 series transferred to cryoprotectant and stored in at −20 °C.

To minimize variance due to immunohistochemical assay procedures, tissue samples were combined such that each contained the brain sections from 2 rats: one treated with MPH and one with dH_2_O of the same strain, from each group. A nick in both hemispheres of the brains of the MPH treated rats distinguished the sections. 

The tissue to be reacted for immunohistochemistry were washed in 5× saline-sodium citrate buffer with 0.1% Tween 20 followed by incubation at 58 °C overnight with gentle agitation. Three 30 min washes at room temperature with Tris-phosphate buffer saline (TPBS) followed. The tissue was incubated in primary antibody solution, consisting of mouse anti-TH antibody (1:5000, Sigma T1299), 10% normal horse serum (NHS) and TPBS containing 0.05% merthiolate (TPBSm)for 1 h at room temperature and then 4 °C for 48 h. Following three 30 min washes in TPBS, the tissue was incubated with secondary antibody, consisting of donkey anti-mouse Cy3 (1:500, Jackson Immunoresearch), 5% NHS and TPBS for 1 h at room temperature and then 4 °C for 12 h. Tissue was washed thrice for 20 min in cold TPBS, mounted on glass slides and coverslipped with Vectashield Hardset (Vector Labs).

#### 4.5.2. Image Analysis

Sections were visualised using an Axiocam MRM camera mounted on a Zeiss Z1 microscope attached to a PC. Using Axiovision software, mosaic images containing 70%–90% of the section were captured at 10× magnification. All microscope and computer settings remained constant to obtain comparable fluorescence of each section. The exposure time was set to 400 ms for each section.

Quantitative optical densitometry of TH staining was performed. The average pixel density (of 65,000 grey levels) of each region of interest (ROI) was determined using a square probe (50,000 µm^2^). Background staining and variations in the illumination levels between images was accounted for by subtracting the mean grey value from a section of tissue (400 µm^2^) with minimal labelling, from the ROI. The adjusted grey value for a ROI was averaged across all the images on which it appeared (minimum of 3 images for each ROI) prior to statistical analysis. 

### 4.6. Statistical Analyses

#### 4.6.1. Statistical Analyses for Behaviour

Analyses were conducted using Analysis of Variance (ANOVA). The General Linear Model was used unless otherwise specified with multivariate statistics reported when the assumption of sphericity was violated. Bonferroni adjustments were used when multiple comparisons were performed. 

Data is presented for each strain separately. The data for the WKYs are presented first as they are the misdiagnosed or non-ADHD strain. The SHR data follows as they act as a control strain.

For analysis of the dose-response curve the General Linear Model was used with Greenhouse-Geisser Epsilon (G-G) adjustments for univariate statistics reported as the assumption of sphericity was violated. A within subjects design was used with four levels of the factor, “dose” (0 mg/kg, 2 mg/kg, 5 mg/kg, 10 mg/kg) and seven levels of the factor, “time” (baseline plus half hourly intervals for 3 h). Contrasts were planned to compare the 0 mg/kg dose to each of the 2 mg/kg, 5 mg/kg, and 10 mg/kg doses with bonferroni adjustments made for multiple comparisons of dose (*p* = 0.017).

A repeated measures analysis was conducted on the locomotor activity measured on the first day of each week of treatment. The analysis had 2 within subjects factors, with 4 levels of “week” (week 1, week 2, week 3, and week 4) and 5 levels of “time” (baseline, 15 min, 30 min, 45 min, and 60 min), and 2 levels of the between subjects factor, “treatment” (MPH *vs.* dH_2_O). There were 12 WKYs in each treatment group. Two rats were removed from the SHR analysis as their performance was ±2 s.d. from the mean, leaving 9 SHRs treated with MPH and 12 SHRs treated with dH_2_O.

Data for the DR task was analysed by a separate repeated measures analysis for each test. The analysis included 5 levels of the within subjects factor, “delay” (five different durations depending on the test, Table 1) and 2 levels of the within subjects factor, “treatment” (MPH *vs.* dH_2_O). Rats from each group and strain were removed from the analysis if their response for that test was ±2 s.d. from the mean. A single SHR in the dH_2_O group failed to learn the task and was removed from all analyses. 

Repeated measures analysis assessed the number of immediate and delayed level presses at 20 s intervals over the 5 min EXT task. There were 2 within subjects factors, with 2 levels of the factor, “lever” (immediate and delayed) and 15 levels of the factor, “time” (20 s intervals over 5 min), and 2 levels of the between subject factor, “treatment” (MPH *vs.* dH_2_O). All animals that completed the DR task, completed the EXT task. Due to a computer malfunction data was lost for a WKY treated with MPH resulting in final group numbers of: *n* = 11 WKY MPH, *n* = 12 WKY dH_2_O and *n* = 11 in each treatment for SHRs.

#### 4.6.2. Statistical Analyses for Immunohistochemistry

The General Linear Model was used to compare the adjusted grey value of each ROI (orbiotofrontal cortex (OFC), prelimbic cortex (PrL), and infralimbic cortex (IL)) between treatment and group factors. Separate analyses were conducted for each ROI with 2 levels of the between subjects factor “treatment” (MPH *vs.* dH_2_O), and 3 levels of the between subjects factor “group” (short-term *vs.* long-term *vs.* behavioural). Fisher’s least significant difference adjustments were used when multiple comparisons were performed.

## 5. Conclusion

In conclusion, the results of this study suggest there are elevated levels of impulsive choice in adulthood when “normal” rats inappropriately received chronic MPH treatment throughout adolescence and such treatment may interfere with the development of catecholamine projections within the PFC, in the WKY strain only. However, when chronic MPH treatment was appropriately given to an animal model of ADHD, there were no long-term effects observed in adulthood. Such a finding has particularly relevance for children that are misdiagnosed with ADHD and as a result, are chronically medicated with psychostimulants such as MPH. It could be inferred from the results of this study that children misdiagnosed with ADHD may have enduring deficits in adulthood as a result of their treatment. This study highlights the importance of developing more sensitive, less subjective diagnostic criteria for ADHD. 
